# Hybrid high-definition microvessel imaging/shear wave elastography improves breast lesion characterization

**DOI:** 10.1186/s13058-022-01511-5

**Published:** 2022-03-05

**Authors:** Juanjuan Gu, Redouane Ternifi, Nicholas B. Larson, Jodi M. Carter, Judy C. Boughey, Daniela L. Stan, Robert T. Fazzio, Mostafa Fatemi, Azra Alizad

**Affiliations:** 1 Department of Physiology and Biomedical Engineering, Mayo Clinic College of Medicine and Science, 200 First Street SW, Rochester, MN 55905 USA; 2 Department of Quantitative Health Sciences, Mayo Clinic College of Medicine and Science, Rochester, MN 55905 USA; 3 Department of Laboratory Medicine and Pathology, Mayo Clinic College of Medicine and Science, Rochester, MN 55905 USA; 4 Department of Surgery, Mayo Clinic College of Medicine and Science, Rochester, MN 55905 USA; 5 Department of Medicine, Mayo Clinic College of Medicine, Rochester, MN 55905 USA; 6 Department of Radiology, Mayo Clinic College of Medicine and Science, Rochester, MN 55905 USA

**Keywords:** Breast cancer diagnosis, Shear wave elastography, Hybrid high-definition microvessel imaging, Clinical factors, SWE-HDMI-C detection model, Improved diagnosis accuracy

## Abstract

**Background:**

Low specificity in current breast imaging modalities leads to increased unnecessary follow-ups and biopsies. The purpose of this study is to evaluate the efficacy of combining the quantitative parameters of high-definition microvasculature imaging (HDMI) and 2D shear wave elastography (SWE) with clinical factors (lesion depth and age) for improving breast lesion differentiation.

**Methods:**

In this prospective study, from June 2016 through April 2021, patients with breast lesions identified on diagnostic ultrasound and recommended for core needle biopsy were recruited. HDMI and SWE were conducted prior to biopsies. Two new HDMI parameters, Murray’s deviation and bifurcation angle, and a new SWE parameter, mass characteristic frequency, were included for quantitative analysis. Lesion malignancy prediction models based on HDMI only, SWE only, the combination of HDMI and SWE, and the combination of HDMI, SWE and clinical factors were trained via elastic net logistic regression with 70% (360/514) randomly selected data and validated with the remaining 30% (154/514) data. Prediction performances in the validation test set were compared across models with respect to area under the ROC curve as well as sensitivity and specificity based on optimized threshold selection.

**Results:**

A total of 508 participants (mean age, 54 years ± 15), including 507 female participants and 1 male participant, with 514 suspicious breast lesions (range, 4–72 mm, median size, 13 mm) were included. Of the lesions, 204 were malignant. The SWE-HDMI prediction model, combining quantitative parameters from SWE and HDMI, with AUC of 0.973 (95% CI 0.95–0.99), was significantly higher than the result predicted with the SWE model or HDMI model alone. With an optimal cutoff of 0.25 for the malignancy probability, the sensitivity and specificity were 95.5% and 89.7%, respectively. The specificity was further improved with the addition of clinical factors. The corresponding model defined as the SWE-HDMI-C prediction model had an AUC of 0.981 (95% CI 0.96–1.00).

**Conclusions:**

The SWE-HDMI-C detection model, a combination of SWE estimates, HDMI quantitative biomarkers and clinical factors, greatly improved the accuracy in breast lesion characterization.

**Supplementary Information:**

The online version contains supplementary material available at 10.1186/s13058-022-01511-5.

## Background

Breast ultrasound (US) is commonly used in the evaluation of breast lesions. However, its low specificity leads to a significant number of benign biopsies [1]. Addition of US elastography techniques, including shear wave elastography (SWE), provides a relative increase in specificity with US for breast cancer detection [2–5]. However, stiffness is not always a good predictor of malignancy due to poor shear wave propagation in very stiff lesions and leads to false negatives for stiffness on SWE [6]. Moreover, not all cancers are stiff [7] and not every stiff lesion is cancer [8]. Consequently, imaging approaches with incremental predictive value for tumor proliferation and aggressiveness are of great importance [9].

Angiogenesis is essential in local tumor growth and distant metastasis in breast cancer [10]. Invasive breast cancer is angiogenesis-dependent and the extent of angiogenesis can be used as a prognostic factor [11]. There is a statistically significant correlation between microvessel density and tumor histological grade [12]. Moreover, microvessel morphology and its distribution pattern vary between benign and malignant breast tumors, with malignant lesions tending to have more permeable and tortuous vessels [13].

Though Doppler US has the potential to help distinguish malignant from benign tumors [14], it is only sensitive to fast flows, revealing highly fragmented and patchy images of larger vessels and obscuring structural analysis of microvessels. Imaging modalities, such as photoacoustic imaging [15], acoustic angiography [16], ultrasound localization microscopy [17], and most recently, imaging tumor vasculature at super-resolution scales, have been investigated [18]. However, photoacoustic imaging may have limitations for deeper lesions. The requirement for injection of contrast agents in the latter technologies may be inconvenient and costly.

Recently, a quantitative high-definition microvasculature imaging (HDMI) approach was developed [19] to visualize submillimeter vessels as small as 300 µm in diameter. The HDMI technique is based on contrast-free ultrafast ultrasound imaging that includes vessel enhancement and morphological filtering as well as quantification of vessel morphological structures [19, 20]. Various quantitative microvasculature morphological parameters could be obtained with the HDMI technique [20].

Previous studies combined SWE or strain elastography and color Doppler US to improve the accuracy of breast cancer diagnosis [21–24]. In this paper, the morphological information of tumor microvessels obtained by HDMI was first combined with SWE parameters and clinical factors to detect and characterize breast cancer. We hypothesized that the morphological parameters of tumor microvessels are independent from the elasticity parameters, and their information would complement each other in breast lesion characterization. The purpose of this study was to evaluate the efficacy of combining the quantitative parameters of high-definition microvasculature imaging (HDMI) and 2D shear wave elastography (SWE) with clinical factors for improving breast lesion differentiation.

## Methods

### Participants

**Fig. 1:**
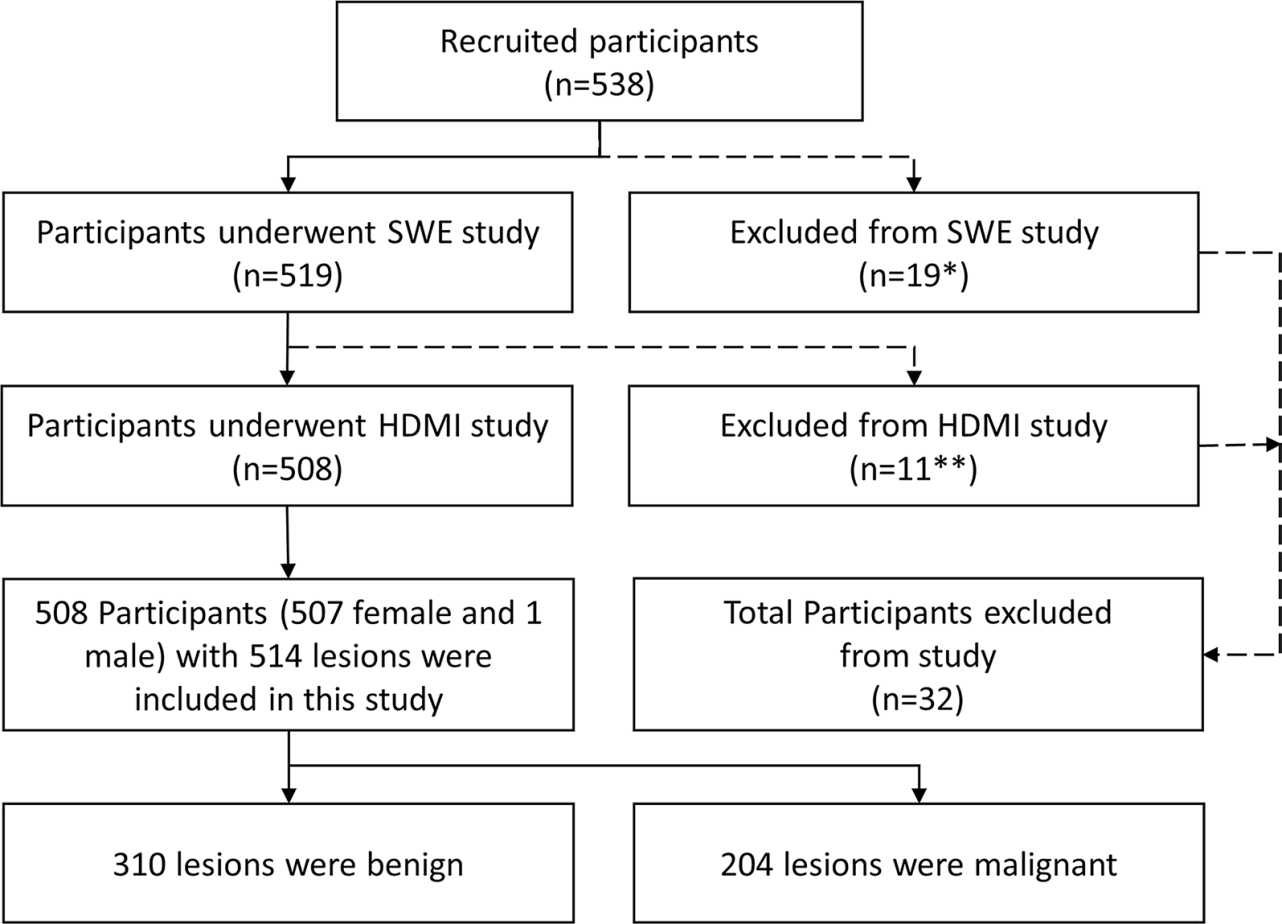
Flowchart for the participants. *19 participants were excluded from the SWE study because the SWE study was cancelled due to insufficient time before biopsy. **11 participants were excluded from the HDMI study (1 was excluded because the scanned lesion was not biopsied, 10 were excluded because of technical problems with the system). HDMI: high-definition microvasculature imaging. SWE: shear wave elastography

This prospective study was approved by our institutional review board (IRB#: 12–003,329 and IRB#: 19–003,028) and was Health Insurance Portability and Accountability Act-compliant. From June 1, 2016 through April 1, 2021, 538 participants with suspicious ultrasound-identified breast lesions and with recommendation for biopsy were recruited for this study. As expected, most cases were classified as BI-RADS 4 and 5; however, three of BI-RADS 3 patients included in this study underwent the biopsy due to patient preference. Volunteers with breast implants or prior mastectomy were excluded during recruitment. Among them, participants who underwent a SWE study only or HDMI study only were excluded from this study. Finally, 508 participants (mean age, 54 years ± 15), including 507 female participants and 1 male participant, with 514 suspicious breast lesions were included in this study. A flowchart for the participant exclusions is shown in Fig. 1. A signed written IRB-approved informed consent with permission for publication was obtained from each enrolled participant prior the study. For all the participants, the pathology results from biopsies served as the reference gold standard and both the SWE study and HDMI study were conducted prior to the biopsy.

### SWE

The US examinations were performed by two sonographers with more than 30/11 and 15/11 years of US/SWE scanning experience, respectively. We used a GE LOGIQ E9 (LE9) clinical scanner equipped with SWE capability and a 9L-D linear array transducer (GE Healthcare, Wauwatosa, WI). The sonographer was instructed to minimize the preload as to reduce artifactual stiffness [25]. In a rectangle-shaped field of view, at least six images were obtained from each lesion. One of the images with the fewest artifacts was processed. Between one and three 3-mm-diameter non-overlapping regions of interests (ROIs) were placed on the stiffest position. The mean shear wave velocity (SWV), maximum SWV, and minimum SWV for each ROI were calculated by the LE9 system. The averaged SWV values of the ROIs were calculated and converted to elasticity in kilopascals for analysis (*E* = 3*ρV*^2^, where *ρ* is tissue density and is 1000 kg/m^3^ in this study, *V* is shear wave velocity) and are listed in the (see Additional file 1: Table S1). *E*_mean_ and *E*_max_ represent the mean and maximum shear wave elasticity, respectively. Additionally, mass characteristic frequency (*f*_mass_ = *V*_min_*/s,* where *V*_min_ is the minimum SWV in m/s and *s* is the mass diameter in m), which was introduced in previous studies [26, 27], was also included in the analysis.

### HDMI

An ALPINION Ecube12-R ultrasound machine, with high-frame-rate imaging capability (ALPINION Medical Systems, Seoul, South Korea) with a linear array transducer L3-12H (ALPINION Medical Systems) operating at 8.5 MHz was used. After identifying breast lesions on plane-wave B-mode ultrasound, a sequence of high frame rate data (~ 600 frames per second) was acquired at the lesion site for a total duration of 3 s. The acquired raw data were processed using the HDMI method, described in detail by Bayat et al. and Demené et al. [19, 28].

Morphological parameters [20, 29], including number of vessel (*NV*), number of branch points (*NB*), vessel density (*VD*), vessel diameter (*D*) and distance metric (*τ*), were measured. Moreover, four new parameters, microvessel fractal dimension (*mvFD*) [29–31]. Murray’s deviation (*MD*) [29, 32–34], bifurcation angle (*BA*) [29, 35–37], and spatial vascular pattern (*SVP*) [29, 38], calculated by vessel density ratio (*VDR*) [29, 32, 34, 39], were also extracted from the HDMI image for characterizing the tumor microvessels. Microvessel morphometric analysis is shown in Appendix 1. Definition of HDMI parameters are detailed in the (see Additional file 1: Table S1).

### Clinical ultrasound and pathologic data

Mass size (*s*) and lesion depth (*d*), measured from B-mode ultrasound, are shown in Table 1. Histologic subtype, histologic grade, estrogen receptor (ER), progesterone receptor (PR), human epidermal growth factor receptor (HER2) status, and Ki-67 proliferative index were obtained from biopsy reports. Per ASCO/CAP guidelines [40], immunohistochemical HER2 scores of 0 and 1 + were considered negative and a score of 3 + was considered positive. Equivocal HER2 immunostaining (HER2 score of 2 +) underwent fluorescence in situ hybridization testing for HER2 amplification and was classified per ASCO/CAP guidelines.

**Table 1:** Participant demographics, lesion characteristics, and the summary of quantitative parameters

	Benign (N = 310)	Malignant (N = 204)	*p v*alue^c^
Sex			
Female^a^	306	200	na
Male^a^	0	1	na
Age (y)	49 ± 15	62 ± 13	< 0.001
*s* (mm)	14.5 ± 8.7	18.5 ± 12.9	< 0 .001
*d* (mm)	10.0 ± 6.0	11.0 ± 5.5	.01
SWE parameters			
* E*_mean_ (kPa)	24.8 ± 20.4	79.2 ± 37.5	< 0.001
* E*_max_ (kPa)	50.5 ± 39.8	159.1 ± 67.1	< 0.001
* E*_min_ (kPa)	11.7 ± 10.5	31.0 ± 22.2	< 0.001
* E*_sd_ (kPa)	0.8 ± 0.8	2.6 ± 1.7	< 0.001
* f*_mass_ (Hz)	157.8 ± 94.1	220.0 ± 130.6	< 0.001
HDMI parameters			
* NV*	4.6 ± 8.1	15.6 ± 19.8	< 0.001
* NB*	2.0 ± 4.7	7.8 ± 12.1	< 0.001
* VD*	0.03 ± 0.03	0.05 ± 0.04	< 0.001
* SVP*	0 (254/310)^b^	1 (113/204)^b^	< 0.001
* D*_max_ (mm)	429.9 ± 272.3	617.1 ± 228.0	< 0.001
* D*_mean_ (mm)	343.3 ± 210.0	428.6 ± 133.1	< 0.001
* τ*_max_	1.1 ± 0.3	1.2 ± 0.2	< 0.001
* τ*_mean_	1.03 ± 0.04	1.04 ± 0.04	< 0.001
* BA*_max_ (°)	141.6 ± 52.9	103.4 ± 58.1	< 0.001
* BA*_mean_ (°)	134.1 ± 55.0	80.5 ± 44.4	< 0.001
* MD*_max_	0.22 ± 0.29	0.65 ± 0.28	< 0.001
* MD*_mean_	0.15 ± 0.19	0.45 ± 0.28	< 0.001
* mvFD*	0.91 ± 0.38	1.18 ± 0.21	< 0.001

Unless otherwise specified, numbers are mean ± standard deviation

HDMI, high-definition microvasculature imaging. SWE, shear wave elastography

ªData are numbers of participants

^b^Numbers are percentages

^c^Kruskal-Wallis test

### Statistical analysis

Quantitative values were summarized by mean ± standard deviation, while categorical variables were summarized as counts and percentages. Testing for distributional differences among the quantitative parameters by lesion malignancy status and tumor characteristics was performed using the Kruskal–Wallis test, using the pathology results as the gold standard. Multivariable prediction models for lesion malignancy status were trained via elastic-net logistic regression using R package glmnet. Training was performed using a random selection of 70% (360/514) of lesions, while the remaining 30% (154/514) were used for model validation. Participants with two lesions were relegated to the training set to ensure sample independence for performance evaluation. Penalty parameter tuning was performed using tenfold cross-validation within the training set, with alpha fixed at 0.5. Model discrimination was evaluated in the independent test set using receiver operating characteristic (ROC) curves based on predicted malignancy probabilities, while optimal threshold selection for discrimination was determined as the point closet to the point (0, 1) on the ROC curve. The probability was calculated with the function: $\mathrm{probability}={\mathrm{logit}}^{-1}(B+\sum_{i=1}^{m}{C}_{m}{P}_{m})$, where $B$ is a constant obtained from the elastic-net logistic regression, ${P}_{m}$ is the quantitative parameter obtained from SWE, HDMI or clinical factor, ${C}_{m}$ is the coefficient for the corresponding quantitative parameter obtained from the elastic-net logistic regression, $m$ is the number of quantitative parameters included in the prediction model, and the logistic function $logi{t}^{-1}$ is defined as ${\mathrm{logit}}^{-1}(\alpha )=1/(1+\mathrm{exp}(-\alpha ))$ [41].

For each model, we estimated the test-set area under the curve (AUC), the corresponding 95% confidence interval (CI), specificity, and sensitivity. Pair-wise comparisons of model performance based on AUC were conducted using DeLong’s test for paired data. All hypothesis testing was conducted under a two-sided alternative, where appropriate, and the test results were considered significant at an alpha level of 0.05. All statistical analyses were performed using RStudio (R version 4.0.4).

## Results

There were 310 benign lesions and 204 malignant lesions included in this study. Lesion size ranged from 4 to 72 mm, and the median size was 13 mm. Table 2 summarizes the demographic information for all participants. The corresponding quantitative parameters from SWE and HDMI are also presented. Stiffness (*E*_max_ and *E*_mean_) values were significantly higher for malignant lesions than for benign lesions. Vessel diameter (*D*_max_, *D*_mean_) and number of vessel segments (*NV*) were significantly larger for malignant lesions than for benign lesions. Also, malignant lesions showed significant abnormalities in microvessel morphological parameters, such as *mvFD* [30], *NB*, *VD*, *τ*_max_, *τ*_mean_, *MD*_max_, *MD*_mean_, *BA*_max_, and *BA*_mean_, highlighting the importance of structural complexity and irregularity of tumor microvessels in addition to increased vessel density.

**Table 2:** Summary of the significant parameters for different malignant grades and molecular subtypes

	*E*_max_ (kPa)	*f*_mass_ (Hz)	*D*_max_ (mm)	*NV*	*mvFD*	*NB*
Histologic type						
Benign (310)						
Fibroadenoma (109)	49.5 ± 33.8	143.0 ± 64.5	448.6 ± 262.8	5.9 ± 9.8	0.92 ± 0.43	2.8 ± 6.2
Benign changes (77)	44.5 ± 38.6	146.4 ± 94.0	356.9 ± 291.6	2.9 ± 5.4	0.83 ± 0.38	1.2 ± 2.6
Fibrocystic changes (30)	48.6 ± 31.9	171.1 ± 104.4	429.3 ± 282.6	4.4 ± 7.9	0.87 ± 0.43	1.7 ± 4.0
Papilloma (28)	51.8 ± 39.2	186.7 ± 88.3	454.0 ± 273.3	3.4 ± 4.2	0.95 ± 0.28	1.3 ± 1.8
PASH (22)	36.3 ± 28.3	139.8 ± 79.6	438.7 ± 247.5	3.1 ± 3.8	0.95 ± 0.27	1.0 ± 1.6
Fat necrosis (18)	97.1 ± 70.6	203.5 ± 184.1	574.9 ± 106.2	3.9 ± 3.0	1.1 ± 0.11	1.5 ± 1.3
Atypical (14)	51.2 ± 34.3	192.3 ± 105.3	303.4 ± 305.7	5.1 ± 9.9	0.75 ± 0.48	2.7 ± 5.6
Duct ectasia (6)	45.9 ± 20.7	231.8 ± 86.7	501.0 ± 270.8	2.5 ± 2.1	0.95 ± 0.19	0.5 ± 0.5
Adenoma (4)	47.9 ± 29.1	99.2 ± 56.2	655.4 ± 165.2	22 ± 22.7	1.28 ± 0.23	10.5 ± 13.0
Others (2)	103.0 ± 95.8	243.9 ± 71.1	708.5 ± 238.7	10 ± 11.3	1.24 ± 0.25	7 ± 8.5
* p* value^a^	.03	.02	.03	.003	.01	.06
Malignant (204)						
IDC (136)	161.6 ± 65.1	212.3 ± 124.9	640.9 ± 197.3	15.2 ± 15.9	1.19 ± 0.21	7.4 ± 9.2
IMC with mixed ductal and lobular features (27)	174.7 ± 68.3	227.8 ± 139.1	593.0 ± 278.2	17.0 ± 18.4	1.16 ± 0.23	8.2 ± 10.1
ILC (24)	168.8 ± 56.2	241.1 ± 128.9	567.6 ± 268.8	14.6 ± 25.0	1.14 ± 0.19	7.3 ± 14.6
DCIS (15)	94.6 ± 74.5	253.3 ± 172.6	503.8 ± 291.7	7.3 ± 5.9	1.11 ± 0.23	3.7 ± 3.4
Other (2)	154.1 ± 48.5	97.2 ± 90.6	811.4 ± 142.3	100 ± 76.4	1.50 ± 0.13	69 ± 46.7
* p* value^a^	.55	.48	.22	.33	.21	.31
Histologic grade						
I (54)	139.1 ± 69.2	278.5 ± 117.1	553.6 ± 244.3	7.5 ± 8.3	1.10 ± 0.20	3.5 ± 4.4
II (92)	168.0 ± 66.0	220.6 ± 140.7	617.7 ± 210.2	15.0 ± 16.3	1.18 ± 0.18	7.3 ± 9.2
III (54)	165.8 ± 64.2	155.6 ± 90.8	673.9 ± 233.5	24.1 ± 28.6	1.24 ± 0.24	12.4 ± 18.3
* p* value^a^	.05	< .001	.02	< .001	< .001	< .001
Molecular subtypes						
Luminal A (76)	155.7 ± 66.4	257.3 ± 120.5	577.0 ± 241.4	10.8 ± 14.2	1.13 ± 0.21	5.2 ± 8.0
Luminal B (HER2-) (58)	176.4 ± 61.4	218.2 ± 139.0	625.5 ± 181.0	15.2 ± 15.7	1.18 ± 0.16	7.4 ± 9.3
Luminal B (HER2 +) (21)	198.4 ± 49.8	136.2 ± 98.4	727.1 ± 155.5	28.6 ± 32.6	1.30 ± 0.13	14.5 ± 21.8
HER2 + (4)	169.1 ± 31.5	95.5 ± 33.2	628.7 ± 149.6	15.8 ± 14.5	1.20 ± 0.18	7.3 ± 6.4
TNBC (20)	131.6 ± 65.4	189.3 ± 85.6	543.2 ± 311.5	13.9 ± 16.3	1.11 ± 0.35	7.3 ± 10.8
* p* value^a^	.005	< .001	.07	.002	.01	.02

Numbers are mean ± standard deviation, numbers in parentheses are numbers of participants

DCIS, ductal carcinoma in situ. IDC, invasive ductal carcinoma. ILC, invasive lobular carcinoma. IMC with mixed ductal and lobular features, invasive mammary carcinoma with mixed ductal and lobular features. PASH, pseudoangiomatous stromal hyperplasia. TNBC, triple negative breast cancer

^a^Kruskal-Wallis test

**Fig. 2:**
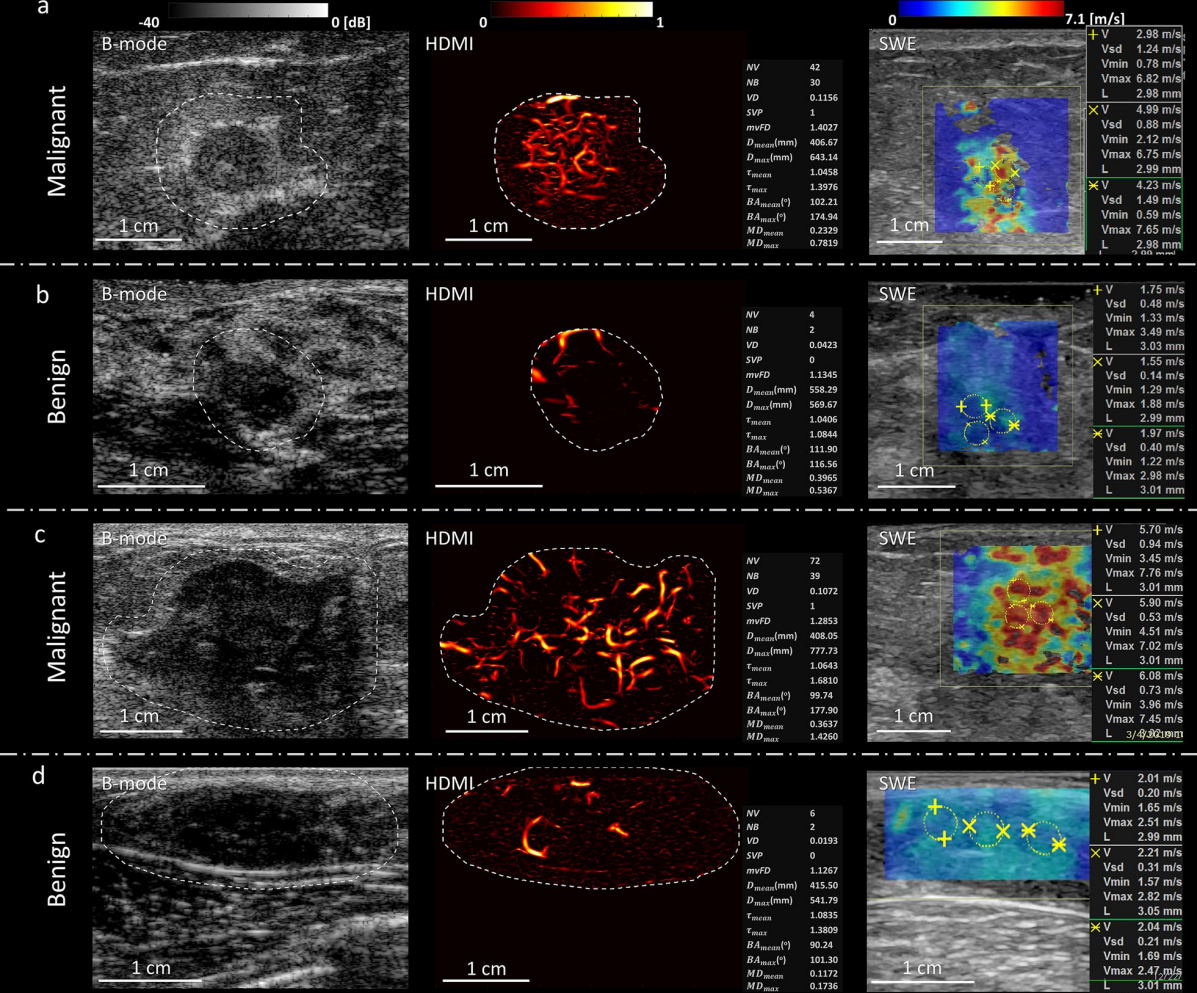
Images and panels of the quantitative parameters of quantitative HDMI and SWE of benign and malignant breast masses grouped by small and large tumor sizes. **a** B-mode, HDMI image and SWE map of a breast tumor of a 71-year-old woman with metastatic breast cancer. Both the quantitate HDMI and the SWE suggest this breast tumor to be malignant. **b** B-mode, HDMI image and SWE map of a breast mass of a 44-year-old woman with fibroadenoma. Both the quantitate HDMI and the SWE suggest this breast mass to be benign. **c** B-mode, HDMI image, and SWE map of a breast tumor of a 63-year-old woman with invasive lobular carcinoma. Both the quantitate HDMI and the SWE suggest this breast tumor to be malignant. **d** B-mode, HDMI image, and SWE map of a fibroadenoma of a 21-year-old woman. Both the quantitative HDMI and the SWE suggest this breast mass to be benign. The dashed-line boundaries in the B-mode and HDMI images represent the lesion border after 2 mm dilatation. HDMI: high-definition microvasculature imaging. SWE: shear wave elastography

Figure 2 summarizes the imaging results from two malignant and two benign breast lesions using the HDMI and SWE methods. Figure 2a and b shows the B-mode, HDMI and SWE image of a small breast cancer with a mass size of 8 mm and a small benign breast lesion with a mass size of 8 mm, respectively. Figure 2c and d shows the B-mode, HDMI and SWE images of a large breast cancer with mass size of 36 mm and a large benign breast lesion with a mass size of 21 mm, respectively. Hypervascularity and morphological irregularity revealed in the HDMI image along with its quantitative parameters, as well as the SWE estimation, correctly suggested these lesions shown in Fig. 2a and c, to be malignant. The presence of only a few regularly formed microvessels in the HDMI image and its quantitative parameters, as well as SWE estimation, shown in Fig. 2b and d, correctly suggested the lesion to be benign.

**Fig. 3:**
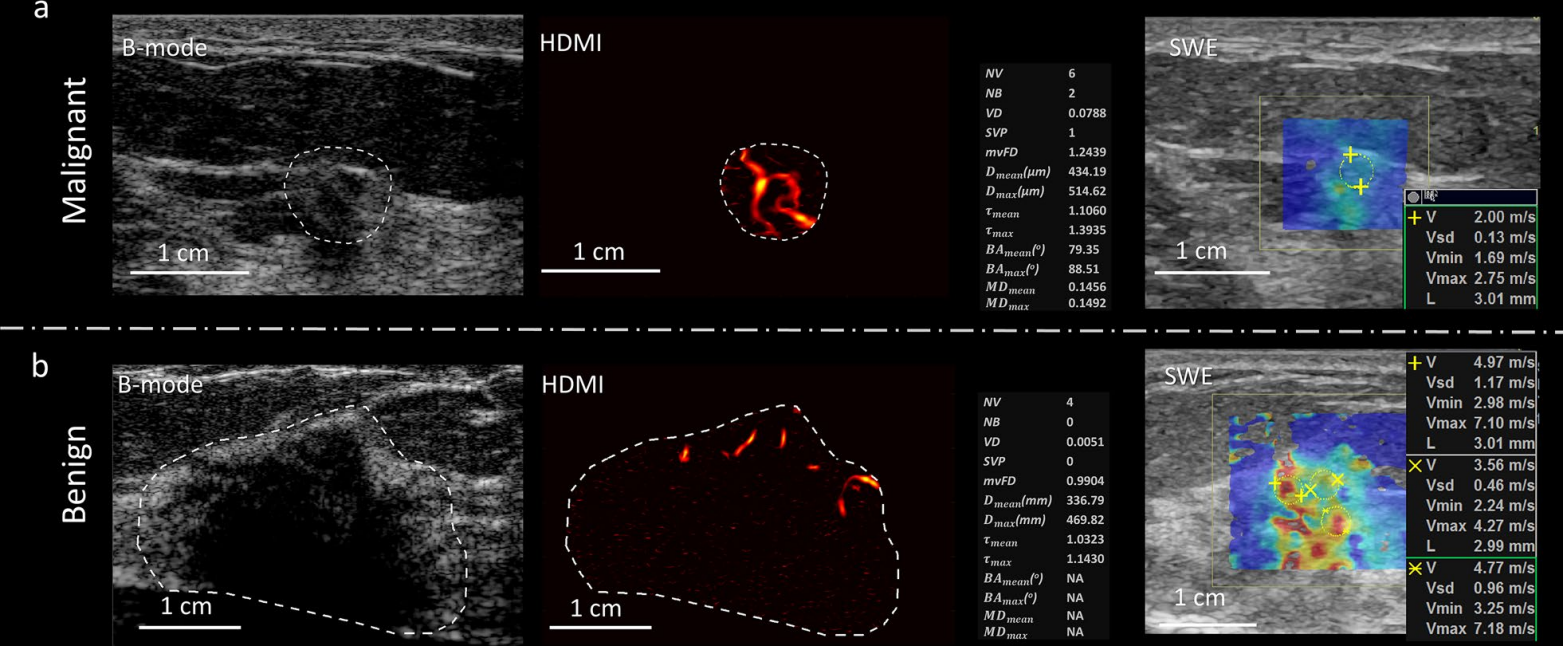
**a** B-mode, HDMI image and SWE map of a breast tumor of a 40-year-old woman with invasive ductal carcinoma. The HDMI image along with the morphological parameters is suggestive of malignancy. SWE map and velocity metrics on the side panel shows low stiffness, incorrectly suggesting the lesion is benign. **b** B-mode, HDMI image and SWE map of a pseudoangiomatous stromal hyperplasia of a 24-year-old woman. HDMI image shows a few microvessels along with the morphological parameters, shown on the side panel, and is suggestive of benign mass. SWE map and velocity metrics on the side panel incorrectly suggest the mass to be malignant. The dashed-line boundaries in the B-mode and HDMI images represent the lesion border after 2 mm dilatation. HDMI: high-definition microvasculature imaging. SWE: shear wave elastography

Figure 3 presents true positive and true negative results of quantitative HDMI of breast tumors of which SWE had false negative and false positive results, respectively. Increased vessel density with morphological irregularity as shown in quantitative parameters of HDMI in Fig. 3a, correctly indicated malignancy for a participant with a breast mass with size 4 mm and a pathology result of invasive ductal carcinoma, where the SWE map for this very small cancer falsely showed low stiffness (false negative with SWE). Furthermore, a few regularly formed vessels, as shown in Fig. 3b, indicated benignity in a participant with breast mass, 20 mm in size, and pathology result of a benign pseudoangiomatous stromal hyperplasia, where the SWE map with high stiffness incorrectly suggested a malignant lesion (false positive with SWE).

### Differentiating malignant lesions from benign lesions with HDMI and SWE individually

**Fig. 4:**
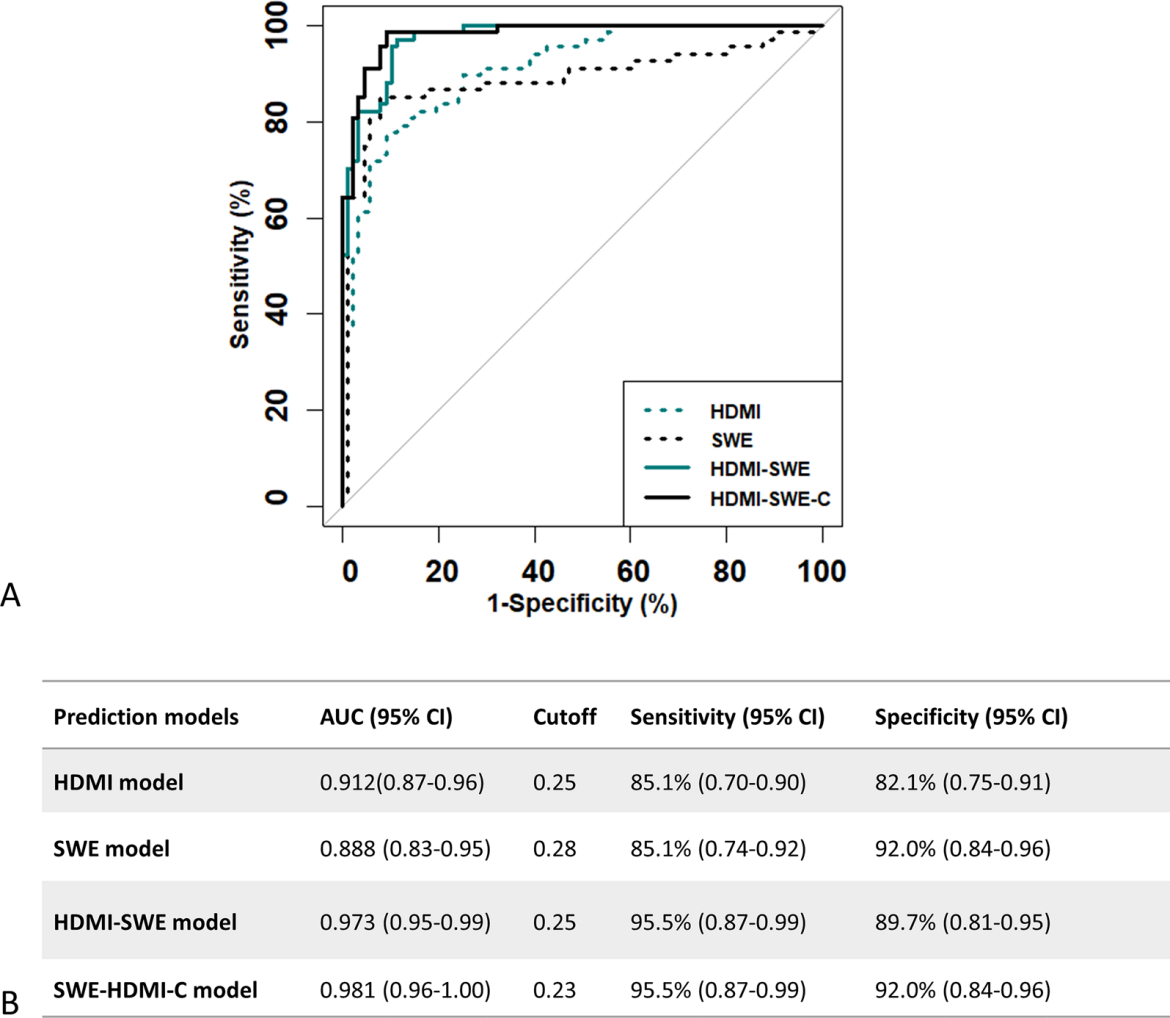
**a** ROC curves generated with the HDMI model (dashed-teal), SWE model (solid-black), HDMI-SWE model (solid-teal) and the HDMI-SWE-C model (solid-black). **b** Summary of the test-set discrimination performance for the lesion diagnosis with competing prediction models. The HDMI model consists of quantitative parameters from HDMI only. The SWE model consists of quantitative parameters from SWE only. The HDMI-SWE model consists of quantitative parameters from both HDMI and SWE. The HDMI-SWE-C model consists of parameters from HDMI, SWE and clinical factors. HDMI:high-definition microvasculature imaging. ROC: receiver operating characteristic. SWE: shear wave elastography

The models developed using parameters from HDMI only and from SWE only were denoted as “HDMI model” and “SWE model,” respectively. The corresponding ROC curves are shown in Fig. 4a. *NV*, *NB*, *VD*, *SVP*, *D*_max_, *D*_min_, *τ*_mean_, *τ*_max_, *BA*_mean_, *BA*_max_, *MD*_mean_, and *FD* were included in the HDMI model. The AUC was 0.912 (95% CI 0.87–0.96). With an optimal cutoff for the malignancy probability at 0.25, the sensitivity and specificity were 82.1% (0.70–0.90) and 85.1% (0.75–0.91), respectively. *E*_mean_, *E*_max_, and *f*_mass_ were included in the SWE. The AUC was 0.888 (95% CI 0.83–0.95). With an optimal cutoff for malignancy probability at 0.28, the sensitivity and specificity were 85.1% (0.74–0.92) and 92.0% (0.84–0.96), respectively.

### Differentiating malignant lesions from benign lesions with the combination of HDMI, SWE, and Clinical factors

HDMI parameters were first combined with the SWE parameters, and the model was denoted as the HDMI-SWE model. *NV*, *NB*, *VD*, *SVP*, *D*_max_, *D*_min_, *τ*_mean_, *τ*_max_, *BA*_mean_, *BA*_max_, *MD*_mean_, *FD*, *E*_mean_, *E*_max_, and *f*_mass_ were included in the HDMI-SWE model. The AUC (0.973, 95% CI 0.95–0.99) was significantly improved when compared to the HDMI model (*p* = 0.001) or the SWE model (*p* = 0.004). With an optimal cutoff for the malignancy probability as 0.25, the sensitivity and specificity were 95.5% (0.87–0.99) and 89.7% (0.81–0.95), respectively.

The AUC was further increased (*p* = 0.32) when clinical factors, including age and *d*, were added to the HDMI-SWE model. The new model was denoted as the HDMI-SWE-C model. The corresponding AUC estimate was 0.981 (95% CI 0.96–1.00) and was the highest among the four models. With an optimal cutoff for malignancy probability at 0.23, the sensitivity and specificity were 95.5% (0.87–0.99) and 92.0% (0.84–0.96), respectively. Table 3 summarizes the true negative, false positive, true positive, and false negative numbers in each BI-RADS category for different prediction models. There were three BI-RADS 3 lesions and 31 BI-RADS 5 lesions. HDMI-SWE and HDMI-SWE-C models successfully classified all lesions in the BI-RADS 3 and 5 categories. There were 120 lesions with BI-RADS 4 assessments. With the HDMI-SWE-C model, 80 benign biopsies were successfully captured. However, 3 of the malignant lesions with BI-RADS score 4 were incorrectly predicted as benign. All three lesions were small, 10 mm in size. The pathology results indicated the first as IDC with Nottingham grade I, the second as IDC with Nottingham grade III and the third as ductal carcinoma in situ (DCIS). Figure 4b summarizes the ROC curves for breast cancer diagnosis with different prediction models. Finally, the percentage of false positive for fat necrosis was 50% (3/6) and the percentage of false negatives for ductal carcinoma in situ was 14.3% (1/7). The practical value for the SWE-HDMI-C model is shown in Appendix 2.

**Table 3:** Summary of the true negative, false positive, true positive, and false negative for the lesions included in the validation set with different prediction models

	Pathology	HDMI	SWE	HDMI-SWE	HDMI-SWE-C
BI-RADS 3					
True negative	3	3	3	3	3
False positive		0	0	0	0
True positive	0	0	0	0	0
False negative		0	0	0	0
BI-RADS 4					
True negative	82	69	75	73	75
False positive		13	7	9	7
True positive	38	31	29	35	35
False negative		7	9	3	3
BI-RADS 5					
True negative	2	2	2	2	2
False positive		0	0	0	0
True positive	29	24	28	29	29
False negative		5	1	0	0

HDMI, high-definition microvasculature imaging. SWE, shear wave elastography

Stiffness for small lesions is underestimated due to the relatively large shear wavelength [42, 43]. However, adding lesion size to the final HDMI-SWE-C prediction model did not improve the model performance. This could be because the HDMI used in this study has been demonstrated to be a more powerful tool for visualizing deep-seated small vessels [19].

### Histologic subtypes and molecular subtypes

Table 2 summarizes the histological subtypes for both the benign and malignant lesions. Among benign subtypes, fat necrosis showed higher stiffness, while the HDMI parameters for fat necrosis, for example, vessel diameter (*D*_max_), number of vessels (*NV*), and number of branches (*NB*), were similar to other benign subtypes but were smaller than the malignant ones.

Based on the clinical biomarker status, invasive carcinomas were divided into five molecular subtypes according to the St. Gallen criteria, as shown in Table 2. Table 2 also summarizes the subset of parameters that were significantly associated with different malignant grades and/or molecular subtypes based on Kruskal–Wallis testing. Higher tumor grade was significantly associated with higher *D*_max_, *NV*, *mvFD*, *NB*, and lower *f*_mass_ values. Less aggressive subtypes, Luminal A and Luminal B (HER2-), corresponded to lower *E*_max_, *D*_max_, *NV*, *NB*, and higher *f*_mass_ values.

Supplementary Video 1 (See Additional file 2: Video S1) illustrates the performance of using *NV*, *FD*, and *f*_mass_ values for differentiating lower tumor cancer grades (grade I/II) from higher grades of cancers (grade III), and the corresponding *p*-values were all less than 0.001. Supplementary Video 2 (see Additional file 3: Video S2,) illustrates the performance of using *NV*, *FD* and *f*_mass_ values for differentiating the less aggressive molecular subtypes (Luminal A and Luminal B (HER2-) subtypes) from other subtypes, and the corresponding *p*-values were 0.002, 0.01 and < 0.001, respectively.

## Discussion

This study investigated the effectiveness of combined quantitative microvessel biomarkers of HDMI and SWE parameters with clinical factors for characterization of breast masses. Our results showed that collective information from independent quantitative parameters of SWE, which measures tumor stiffness, and HDMI, which quantifies tumor angiogenesis, increased the accuracy of differentiation of breast masses with a sensitivity and specificity of 95.5% (0.87–0.99) and 89.7% (0.81–0.95), respectively. When combined with the clinical factors (lesion depth and age) specificity increased to 92.0% (0.84–0.96) and the sensitivity was kept as 95.5% (0.87–0.99). Previous strain imaging using the E/B ratio in a meta-analysis showed a sensitivity of 96% and specificity of 88% [44]. Future studies which include the strain imaging could probably further help increase accuracy in characterizing breast masses.

Combined SWE and color Doppler ultrasound increases the specificity of B-mode US [21–23]; however, structural analysis of microvessels using this technique is not possible, as only larger vessels in a form of fragmented and patchy images are revealed. Further, the combination of SWE and superb microvascular imaging (SMI) using the SMI vascular index has shown a relative increase in specificity [45]. However, the SMI technique is not based on microvessel morphology. Rather, SMI uses a marker derived from pixel count. The HDMI technique used in this study quantifies more than 10 microvessel morphological parameters to characterize the tumor. To achieve this characterization, HDMI uses multi-scale vessel enhancement filtering to extract the tumor microvasculature structures at the submillimeter levels without injecting contrast agents, followed by a series of advanced algorithms to prepare them for quantification. When compared to the other techniques used for characterizing angiogenesis, such as DCE-MRI and contrast-enhanced mammography, the proposed modality is noninvasive, quantitative, fast, low-cost, and portable. The new technique, HDMI**,** does not require injection of high-cost contrast agents, which simplifies its use in routine clinical practice in a truly noninvasive approach.

In this study, two new microvessel morphological parameters obtained from HDMI, bifurcation angle (*BA*) and Murray’s deviation (*MD*), were first used for breast masses differentiation. Our results showed that both *BA* and *MD* were significantly associated with pathology results. Similarly, it has been shown in previous studies that *BA* is smaller in invasive carcinomas of the colon than that in normal tissue [36] and diseased tissue shows a higher *MD *[46]. A new shear wave parameter, *f*_mass_, was also used in this study. *f*_mass_ has previously been shown to be a discriminating biomarker for breast lesion malignancy and the status of various immunochemical biomarkers (ER, PR, HER2 and Ki-67) [26]. The current study showed that this parameter was also significantly associated with the pathology results, with benign lesions demonstrating lower *f*_mass_ values.

In this study, HDMI was able to capture the microvasculature structures in a breast lesion as small as 4 mm. It has been shown that formation of new microvessels toward and within the malignant tumor occurs when a tumor reaches 2–4 mm in size [10]. While HDMI was able to visualize and quantify the microvessels in this very small breast cancer, the SWE map falsely detected the lesion as benign. In this study, 3 small (< 10 mm) malignant lesions with BI-RADS score 4 were incorrectly predicted as benign. This outcome is in agreement with other studies, showing that SWE has lower sensitivity for detecting malignant lesions less than 10 mm in diameter, as SWV is underestimated in small lesions < 10 mm, leading to false negatives [3, 42, 47]. Also, the presence of calcifications inside a mass and precompression effects during ultrasound scanning lead to an apparent higher stiffness [8, 25]. Therefore, addition of quantitative HDMI may help reduce false negatives/positives of SWE and provide more accurate characterization of breast masses.

Vessel density alone may not be a good marker for breast tumor differentiation, because, for example, increased tumor vascularity has been reported in rapidly-growing benign lesions such as fibroadenoma, intraductal papilloma, and variants of adenosis [48]. Herein, quantification of various microvessel morphological features obtained from HDMI, such as vessel diameter, tortuosity, complexity, and branching, would provide valuable information for accurate tumor characterization, and, with the addition of SWE parameters, help distinguish benign from malignant. Conversely, angiogenesis in breast cancer is heterogeneous and a decreased microvessel density at the centers of the malignant breast tumors is commonly seen [49]. In this study, peritumoral distribution of tumor microvessel was determined by *SVP* calculation. Hence, the collective independent information of the two modalities, morphological parameters of tumor microvessel and the SWE parameters, will help accurate diagnosis in such cases.

When age and lesion depth were combined with the SWE and HDMI parameters, we observed a further increase in discrimination performance with the HDMI-SWE-C model. Age is a well-known factor in diagnosis of breast cancer [50]. Lesion depth is an important factor in prediction modeling because the acoustic signal attenuates significantly in deep-seated lesions [51] and, therefore, degrades the ultrasound-based image.

With the proposed HDMI-SWE-C model, three of the malignant lesions were incorrectly predicted as benign, with one Nottingham grade III. This case shows the limitation of the proposed modality, while the proposed model could be useful in clinical decision for upgrading a presumptive BI-RADS 3 lesions to BI-RADS 4 with recommendation for biopsy. As we explain in Appendix 2, to prevent missed cancer cases, a potential downgrading will never happen without considering additional imagining modalities, and certainly will be at the discretion of the radiologist**.** Further validation, refinement, and testing are needed for eliminating the false negatives and improving characterization of breast masses.

There are some limitations in this study. First, the current study was performed in one center only. Future multicenter studies with a larger population will be needed to further validate our findings. Second, participants in this study all had breast lesions with recommendation for biopsy; therefore, they did not represent the screening population. Third, a nonsignificant number of breast cancers are artifactual false negatives due to improper ROI position selections in the SWE study [52]. Fourth, some of the quantitative parameters could be overlooked due to the limitation from 2D microvasculature imaging. Our on-going work with 3D microvasculature imaging would be helpful for overcoming this limitation. Furthermore, in future studies, we will also include patients with suspicious recurrence of breast cancer in the mastectomy site, to determine the capability of ultrasound microvessel imaging for characterization of breast masses in challenging situations of post-surgical/reconstruction changes.

## Conclusions

The combination of HDMI quantitative microvessel morphological parameters and SWE elasticity estimates are synergistic biomarkers for improved breast mass differentiation. Combining HDMI and SWE with clinical factors (lesion depth and age) further increases this accuracy. This study particularly demonstrates the added value of quantitative microvessel morphological parameters as new biomarkers for improving the specificity as well as sensitivity in differentiating benign from malignant lesions.

### Supplementary Information

**Additional file 1.** **Table S1** List of the quantitative parameters. Definition of shear wave elastography and high-definition microvessel imaging parameters are detailed in this table.

**Additional file 2.** 3D animation illustrating the performance of using NV, FD and fmass values for differentiating lower grade cancers (Grade I/II, teal circles) from higher grade cancers (Grade III, red circles). The corresponding regression planes are also shown in the video. See Additional file 2: Video S1 illustrates the performance of using NV, FD and fmass values for differentiating lower grades of cancers (grade I/II) from higher grade cancers (grade III), and the corresponding p-values were all less than 0.001.

**Additional file 3.** 3D animation illustrating the performance of using NV, FD and fmass values for differentiating the less aggressive molecular subtypes (Luminal A and Luminal B (HER2-) subtypes illustrated in teal circles) from other subtypes (red circles). The corresponding regression planes are also shown in the video. See  Additional file 3: Video S2  illustrates the performance of using NV, FD and fmass values for differentiating the less aggressive molecular subtypes (Luminal A and Luminal B (HER2-) subtypes) from other subtypes, and the corresponding p-values were 0.002, 0.01 and <0.001, respectively.

## Data Availability

The data that support the findings of this study are available from the corresponding author upon reasonable request. The requested data may include figures that have associated raw data. Because the study was conducted on human volunteers, the release of patient data may be restricted by Mayo policy and needs special request. The request can be sent to: Karen A. Hartman, MSN, CHRC | Administrator—Research Compliance| Integrity and Compliance Office | Assistant Professor of Health Care Administration, Mayo Clinic College of Medicine & Science | 507–538-5238 | Administrative Assistant: 507–266-6286 | hartman.karen@mayo.edu Mayo Clinic | 200 First Street SW | Rochester, MN 55,905 | mayoclinic.org. We do not have publicly available Accession codes, unique identifiers, or web links.

## Appendix 1

### High-definition microvasculature imaging and vessel extraction

The quantitative HDMI approach is based on three major steps: 1) image formation by recording a large sequence of images from breast tumor at a high frame rate followed by a series of vessel filtering to enhance the vessels, without injection of a contrast-enhancing agent; 2) a set of processing steps for morphological filtering and segmentation that prepares the image for quantification; and 3) quantification of vessel structures using the quantification tools.

### Microvessel morphometric analysis

A ROI was demarcated based on the lesion boundary obtained from the B-mode ultrasound image. To include peripheral vascularity, the entire initial ROI was enlarged by 2 mm. To quantify vessel morphology, vessel skeletons were extracted from the gross microvasculature images to construct the full skeleton [20]: converting the microvasculature image (output of Hessian filter) to a binary image, removing small noise-like objects through an erosion and dilation operation, determining the mid-line of each vessel, and constructing the full skeleton of the vessel network [20]. Finding the skeleton is based on a thinning algorithm [20, 53]. In this approach, vessels are sequentially thinned, and the midline of each vessel is determined to construct the skeleton of a vessel network, which only includes the centerlines of the microvessels [20]. After these steps, the output image includes the vessel segments. These vessel segments are analyzed to estimate the desired quantitative morphological parameters of the vessels, which are well described and detailed by Ghavami et al. and Caresio et al. [20, 29, 38].

## Appendix 2

### Practical value for the proposed SWE-HDMI-C model for characterization of breast masses

The current study included patients with suspicious breast masses who were recommended for breast core needle biopsy. Prior to biopsy, patients participated in the combined SWE and HDMI evaluations. Malignancy probability was subsequently calculated based on the parameters obtained from SWE and HDMI using the SWE-HDMI-C model. With this approach, sensitivity was 95.5% (0.87–0.99) and specificity was 92.0% (0.84–0.96).

With additional validation, refinement and testing, hybrid SWE and HDMI could potentially be utilized as complementary tools to conventional clinical ultrasound to improve characterization of breast masses. The proposed malignancy probability score generated by this approach may add value when determining which findings should be recommended for biopsy. For this purpose, if the malignancy probability score is higher than the cutoff (0.23 in this study), the algorithm would be supportive of breast biopsy. If the malignancy probability score is below the cutoff, the algorithm would be more supportive of follow up. As such, this model could be useful in clinical decision making, potentially downgrading a presumptive BI-RADS 4a lesion to BI-RADS 3 with recommendation for follow-up or upgrading a presumptive BI-RADS 3 lesion to BI-RADS 4 with recommendation for biopsy. Of course, this would be at the discretion of the radiologist.

## Abbreviations

AUC: Area under the curve
CI: Confidence interval
HDMI: Non-contrast-enhanced ultrasound microvasculature imaging
HER2: Human epidermal growth factor receptor
ROC: Receiver operating characteristic
ROI: Region of interest
SMI: Superb microvascular imaging
SWE: Shear wave elastography
SWV: Shear wave velocity
US: Ultrasound

## Code availability

The custom code or mathematical algorithms that are deemed central to the conclusions are available from the corresponding author upon request.

## References

[1] Berg WA, Blume JD, Cormack JB, Mendelson EB, Lehrer D, Böhm-Vélez M, Pisano ED, Jong RA, Evans WP, Morton MJ (2008). Combined screening with ultrasound and mammography vs mammography alone in women at elevated risk of breast cancer. JAMA.

[2] Berg WA, Cosgrove DO, Doré CJ, Schäfer FK, Svensson WE, Hooley RJ, Ohlinger R, Mendelson EB, Balu-Maestro C, Locatelli M (2012). Shear-wave elastography improves the specificity of breast US: the BE1 multinational study of 939 masses. Radiology.

[3] Gu J, Polley EC, Ternifi R, Nayak R, Boughey JC, Fazzio RT, Fatemi M, Alizad A: Individualized-thresholding Shear Wave Elastography combined with clinical factors improves specificity in discriminating breast masses. *The Breast* 2020. 10.1016/j.breast.2020.10.013 PMC7670190 33188991

[4] Denis M, Bayat M, Mehrmohammadi M, Gregory A, Song P, Whaley DH, Pruthi S, Chen S, Fatemi M, Alizad A (2015). Update on breast cancer detection using comb-push ultrasound shear elastography. IEEE Trans Ultrason Ferroelectr Freq Control.

[5] Bayat M, Denis M, Gregory A, Mehrmohammadi M, Kumar V, Meixner D, Fazzio RT, Fatemi M, Alizad A: Diagnostic features of quantitative comb-push shear elastography for breast lesion differentiation. *PloS one* 2017, 12(3):e0172801. 10.1371/journal.pone.0172801 PMC5336209 28257467

[6] Barr RG, Zhang Z (2015). Shear-wave elastography of the breast: value of a quality measure and comparison with strain elastography. Radiology.

[7] Kim SJ, Ko KH, Jung HK, Kim H. Shear wave elastography: is it a valuable additive method to conventional ultrasound for the diagnosis of small (≤ 2 cm) breast cancer? Medicine. 2015;94(42):e1540. 10.1097/MD.0000000000001540 PMC4620821 26496257

[8] Gregory A, Mehrmohammadi M, Denis M, Bayat M, Stan DL, Fatemi M, Alizad A. Effect of calcifications on breast ultrasound shear wave elastography: an investigational study. PLoS One. 2015;10(9)::e0137898. 10.1371/journal.pone.0137898 PMC4569403 26368939

[9] Son MJ, Kim S, Jung HK, Ko KH, Koh JE, Park AY (2020). Can ultrasonographic vascular and elastographic features of invasive ductal breast carcinoma predict histologic aggressiveness?. Acda Radiol.

[10] Weidner N (1995). Current pathologic methods for measuring intratumoral microvessel density within breast carcinoma and other solid tumors. Breast Cancer Res Treat.

[11] Uzzan B, Nicolas P, Cucherat M, Perret G-Y (2004). Microvessel density as a prognostic factor in women with breast cancer: a systematic review of the literature and meta-analysis. Can Res.

[12] Raman D, Boj S, Arumugam D, Chidambaram L (2017). An assessment of angiogenesis in fibrocystic breast disease and invasive breast carcinoma. J Evol Med Dent Sci.

[13] Du J, Li F-H, Fang H, Xia J-G, Zhu C-X (2008). Microvascular architecture of breast lesions: evaluation with contrast-enhanced ultrasonographic micro flow imaging. J Ultrasound Med.

[14] Raza S, Baum JK (1997). Solid breast lesions: evaluation with power Doppler US. Radiology.

[15] Yamaga I, Kawaguchi-Sakita N, Asao Y, Matsumoto Y, Yoshikawa A, Fukui T, Takada M, Kataoka M, Kawashima M, Fakhrejahani E (2018). Vascular branching point counts using photoacoustic imaging in the superficial layer of the breast: A potential biomarker for breast cancer. Photoacoustics.

[16] Gessner RC, Aylward SR, Dayton PA (2012). Mapping microvasculature with acoustic angiography yields quantifiable differences between healthy and tumor-bearing tissue volumes in a rodent model. Radiology.

[17] Errico C, Pierre J, Pezet S, Desailly Y, Lenkei Z, Couture O, Tanter M (2015). Ultrafast ultrasound localization microscopy for deep super-resolution vascular imaging. Nature.

[18] Christensen-Jeffries K, Couture O, Dayton PA, Eldar YC, Hynynen K, Kiessling F, O'Reilly M, Pinton GF, Schmitz G, Tang M-X (2020). Super-resolution ultrasound imaging. Ul Trasound Med Biol.

[19] Bayat M, Fatemi M, Alizad A (2018). Background removal and vessel filtering of noncontrast ultrasound images of microvasculature. IEEE Trans Biomed Eng.

[20] Ghavami S, Bayat M, Fatemi M, Alizad A (2020). Quantification of morphological features in non-contrast-enhanced ultrasound microvasculature imaging. IEEE Access.

[21] Choi JS, Han B-K, Ko EY, Ko ES, Shin JH, Kim GR (2016). Additional diagnostic value of shear-wave elastography and color Doppler US for evaluation of breast non-mass lesions detected at B-mode US. Eur Radiol.

[22] Lee SH, Chung J, Choi HY, Choi SH, Ryu EB, Ko KH, Koo HR, Park JS, Yi A, Youk JH (2017). Evaluation of screening US–detected breast masses by combined use of elastography and color Doppler US with B-mode US in women with dense breasts: a multicenter prospective study. Radiology.

[23] Cho N, Jang M, Lyou CY, Park JS, Choi HY, Moon WK (2012). Distinguishing benign from malignant masses at breast US: combined US elastography and color Doppler US—influence on radiologist accuracy. Radiology.

[24] Li L, Zhou X, Zhao X, Hao S, Yao J, Zhong W, Zhi H (2017). B-mode ultrasound combined with color Doppler and strain elastography in the diagnosis of non-mass breast lesions: A prospective study. Ul Trasound Med Biol.

[25] Barr RG, Zhang Z (2012). Effects of precompression on elasticity imaging of the breast: development of a clinically useful semiquantitative method of precompression assessment. J Ultrasound Med.

[26] Gu J, Polley EC, Boughey JC, Fazzio RT, Fatemi M, Alizad A: Prediction of Invasive Breast Cancer Using Mass Characteristic Frequency and Elasticity in Correlation with Prognostic Histologic Features and Immunohistochemical Biomarkers. *Ul Trasound Med Biol* 2021. 10.1016/j.ultrasmedbio.2021.03.039 PMC8243825 33994231

[27] Gu J, Polley EC, Denis M, Carter JM, Pruthi S, Gregory AV, Boughey JC, Fazzio RT, Fatemi M, Alizad A (2021). Early assessment of shear wave elastography parameters foresees the response to neoadjuvant chemotherapy in patients with invasive breast cancer. Breast Cancer Res.

[28] Demené C, Deffieux T, Pernot M, Osmanski B-F, Biran V, Gennisson J-L, Sieu L-A, Bergel A, Franqui S, Correas J-M (2015). Spatiotemporal clutter filtering of ultrafast ultrasound data highly increases Doppler and fUltrasound sensitivity. IEEE Trans Med Imaging.

[29] Ternifi R WY, Polley EC, Fazzio RT, Fatemi M, Alizad A: Quantitative biomarkers for cancer detection using contrast-free ultrasound high-definition microvessel imaging: fractal dimension, murray’s deviation, bifurcation angle & spatial vascularity pattern. IEEE Trans Med Imaging 2021:1–10. 10.1109/TMI.2021.3101669 PMC8668387 34329160

[30] Sabo E, Boltenko A, Sova Y, Stein A, Kleinhaus S, Resnick MB (2001). Microscopic analysis and significance of vascular architectural complexity in renal cell carcinoma. Clin Cancer Res.

[31] Chen C (2018). He Z-c, Shi Y, Zhou W, Zhang X, Xiao H-l, Wu H-b, Yao X-h, Luo W-c, Cui Y-h: Microvascular fractal dimension predicts prognosis and response to chemotherapy in glioblastoma: an automatic image analysis study. Lab Invest.

[32] Secomb TW, Dewhirst MW, Pries AR (2012). Structural adaptation of normal and tumour vascular networks. Basic Clin Pharmacol Toxicol.

[33] McAllister A, Abramoff M, Xu X (2013). Deviation from the optimal branching relationship of retinal vessels in diabetes mellitus. Invest Ophthalmol Vis Sci.

[34] Taber LA, Ng S, Quesnel AM, Whatman J, Carmen CJ (2001). Investigating Murray's law in the chick embryo. J Biomech.

[35] Ziyrek M, Sertdemir AL, Duran M (2020). Effect of coronary artery bifurcation angle on atherosclerotic lesion localization distance to the bifurcation site. J Saudi Heart Assoc.

[36] Konerding M, Fait E, Gaumann A (2001). 3D microvascular architecture of pre-cancerous lesions and invasive carcinomas of the colon. Br J Cancer.

[37] Kipli K, Hoque ME, Lim LT, Mahmood MH, Sahari SK, Sapawi R, Rajaee N, Joseph A: A review on the extraction of quantitative retinal microvascular image feature. Comput Math Method M 2018, 2018. 10.1155/2018/4019538 PMC6051289 30065780

[38] Caresio C, Caballo M, Deandrea M, Garberoglio R, Mormile A, Rossetto R, Limone P, Molinari F (2018). Quantitative analysis of thyroid tumors vascularity: a comparison between 3-D contrast-enhanced ultrasound and 3-D Power Doppler on benign and malignant thyroid nodules. Med Phys.

[39] Liu H, Jiang Y, Dai Q, Zhu Q, Wang L, Lu J (2014). Peripheral enhancement of breast cancers on contrast-enhanced ultrasound: correlation with microvessel density and vascular endothelial growth factor expression. Ul Trasound Med Biol.

[40] Wolff AC, Hammond MEH, Schwartz JN, Hagerty KL, Allred DC, Cote RJ, Dowsett M, Fitzgibbons PL, Hanna WM, Langer A (2007). American Society of Clinical Oncology/College of American Pathologists guideline recommendations for human epidermal growth factor receptor 2 testing in breast cancer. Arch Pathol Lab Med.

[41] Jordan MI: Why the logistic function? A tutorial discussion on probabilities and neural networks. In*.*: Computational cognitive science technical report; 1995.

[42] Zhang Y, Li G-Y, Zhou J, Zheng Y, Jiang Y-X, Liu Y-L, Zhang L-L, Qian L-X, Cao Y: Size effect in shear wave elastography of small solid tumors–A phantom study. *Extreme Mech Lett* 2020:100636.

[43] Song P, Macdonald MC, Behler RH, Lanning JD, Wang MH, Urban MW, Manduca A, Zhao H, Callstrom MR, Alizad A (2015). Two-dimensional shear-wave elastography on conventional ultrasound scanners with time-aligned sequential tracking (TAST) and comb-push ultrasound shear elastography (CUSE). IEEE T Ul Transon Ferr.

[44] Barr RG, De Silvestri A, Scotti V, Manzoni F, Rebuffi C, Capittini C, Tinelli C (2019). Diagnostic performance and accuracy of the 3 interpreting methods of breast strain elastography: a systematic review and meta-analysis. J Ultrasound Med.

[45] Lee EJ, Chang Y-W (2020). Combination of quantitative parameters of shear wave elastography and superb microvascular imaging to evaluate breast masses. Korean J Radiol.

[46] Xuan R, Zhao X, Jian J, Hu D, Qin L, Lv W, Hu C: Phase-contrast computed tomography: A correlation study between portal pressure and three dimensional microvasculature of ex vivo liver samples from carbon tetrachloride-induced liver fibrosis in rats. Microvasc Res 2019, 125:103884. 10.1016/j.mvr.2019.103884 31176686

[47] Yoon JH, Jung HK, Lee JT, Ko KH (2013). Shear-wave elastography in the diagnosis of solid breast masses: what leads to false-negative or false-positive results?. Eur Radiol.

[48] Zhang X-Y, Zhang L, Li N, Zhu Q-L, Li J-C, Sun Q, Wang H-Y, Jiang Y-X (2019). Vascular index measured by smart 3-D superb microvascular imaging can help to differentiate malignant and benign breast lesion. Cancer Manag Res.

[49] Li Y-J, Wen G, Wang Y, Wang D-X, Yang L, Deng Y-J, Wei H-Q, He J, Zhang X, Gu Y-S (2013). Perfusion heterogeneity in breast tumors for assessment of angiogenesis. J Ultrasound Med.

[50] Thomas G, Leonard R (2009). How age affects the biology of breast cancer. Clin Oncol.

[51] Shin HJ, Kim M-J, Kim HY, Roh YH, Lee M-J (2016). Comparison of shear wave velocities on ultrasound elastography between different machines, transducers, and acquisition depths: a phantom study. Eur Radiol.

[52] Youk JH, Son EJ, Gweon HM, Han KH, Kim J-A: Quantitative lesion-to-fat elasticity ratio measured by shear-wave elastography for breast mass: Which area should be selected as the fat reference? *PLoS One* 2015, 10(9):e0138074. 10.1371/journal.pone.0138074 PMC4569433 26368920

[53] Lam L, Lee SW, Suen CY (1992). Thinning methodologies - a comprehensive survey. Ieee T Pattern Anal.

