# Supplementary material for: Hybrid high-definition microvessel imaging/shear wave elastography improves breast lesion characterization
Source: Breast Cancer Res. 2022 Mar 5;24:16. doi: 10.1186/s13058-022-01511-5 (PMC8898476; doi:10.1186/s13058-022-01511-5)
Supplement: Supplementary file 1 — Additional file 1. Table S1 List of the quantitative parameters. Definition of shear wave elastography and high-definition microvessel imaging parameters are detailed in this table. [file 13058_2022_1511_MOESM1_ESM.docx]

Supplementary Table 1: List of the Quantitative Parameters

| Parameters | Definition |
| --- | --- |
| Clinical factors |  |
| *d* (mm) | Depth: vertical distance from the skin surface to the top of the lesion shown in the B-mode |
| *s* (mm) | Mass size: the largest dimension shown in the B-mode |
| SWE parameters |  |
| *E* (kPa) | Shear wave elasticity (*E_mean_*, *E_max_*) |
| *SWV* (m/s) | Shear wave velocity (*SWV_mean_*, *SWV_max_*) |
| *f_mass_* (Hz) | Ratio of the minimum SWV and the lesion diameter (*f_mass_* = 1000SW*V_min_/s*) |
| HDMI parameters |  |
| *NV* | Number of vessel segments [[20](#_ENREF_20)] |
| *NB* | Numbers of branch points [[20](#_ENREF_20)] |
| *VD* | Vessel density [[20](#_ENREF_20)] |
| *VDR* | Vessel density ratio: ratio of the geometric area of vessel segments to the geometric area of the associated lesion’s region of interest [[38](#_ENREF_38)]. |
| *SVP* | Spatial vascularity pattern: it describes the distribution pattern of microvessels concentrated peripherally (peritumoral vascularization) or inside the lesion (intratumoral vascularization), SVP is calculated by *VDR.* If *VDR < 1*, *SVP = 0*, meaning a more peripherally concentrated vessel distribution. If VAD > 1, *SVP* = 1, meaning a more centrally concentrated vessel distribution [[38](#_ENREF_38)]. |
| *D* (mm) | Vessel diameter (*D_mean_*, *D_max_*): vessel diameter is two times of the minimum distance between the vessel centerline and the vessel border [[20](#_ENREF_20)]. |
| *τ* | Vessel tortuosity metrics (*τ_mean_*, *τ_max_)*: determined by Distance metric (DM_mean_, DM_max_): that measures vascular tortuosity [[20](#_ENREF_20)]. |
| *BA* (°) | Bifurcation angle: Refers to the angle between two daughter vessels. If *NB* = 0, *BA* = 180° [[32-34](#_ENREF_32)]. |
| *MD* | Murray’s deviation (*MD_mean_*, *MD_max_*): diameter mismatch, defined as the deviation from Murray's law, increases in the vasculature network of malignant tumors. If *NB* = 0, *MD* = 1 [[29-31](#_ENREF_29)]. |
| *mvFD* | Microvessel fractal dimension: a unitless geometrical feature that can be calculated by the box counting method to quantify the structural complexity of tumor vessels [[30](#_ENREF_30)]. |

Note ‒ Subscript mean, min and max indicate the corresponding mean, minimum and maximum value, respectively. HDMI, non-contrast-enhanced ultrasound microvasculature imaging. SWE, shear wave elastography.
